# Pulse-modulated multilevel data storage in an organic ferroelectric resistive memory diode

**DOI:** 10.1038/srep24407

**Published:** 2016-04-15

**Authors:** Jiyoul Lee, Albert J. J. M. van Breemen, Vsevolod Khikhlovskyi, Martijn Kemerink, Rene A. J. Janssen, Gerwin H. Gelinck

**Affiliations:** 1 Holst Centre/TNO, High Tech Campus 31, 5656 AE Eindhoven, The Netherlands; 2 Department of Graphic Arts Information Engineering, Pukyong National University, 608-739, Busan, S. Korea; 3 Department of Applied Physics, Eindhoven University of Technology, P. O. Box 513, 5600 MB Eindhoven, The Netherlands; 4 Department of Physics, Chemistry and Biology (IFM), Linköping University, SE-58183, Sweden

## Abstract

We demonstrate multilevel data storage in organic ferroelectric resistive memory diodes consisting of a phase-separated blend of P(VDF-TrFE) and a semiconducting polymer. The dynamic behaviour of the organic ferroelectric memory diode can be described in terms of the inhomogeneous field mechanism (IFM) model where the ferroelectric components are regarded as an assembly of randomly distributed regions with independent polarisation kinetics governed by a time-dependent local field. This allows us to write and non-destructively read stable multilevel polarisation states in the organic memory diode using controlled programming pulses. The resulting 2-bit data storage per memory element doubles the storage density of the organic ferroelectric resistive memory diode without increasing its technological complexity, thus reducing the cost per bit.

There is currently significant research interest in resistive polymer switching driven by the desire for non-volatile, reprogrammable memory for ubiquitous, low-cost computing systems. Ferroelectric polymers such as poly(vinylidene fluoride) (PVDF) and its copolymers with trifluoroethylene (TrFE), P(VDF-TrFE), can be switched by an external electric field between two polarisation states with a relatively large remanent polarisation of 0.05–0.1 C/m^2^. Many groups have studied the ferroelectric properties of these materials in thin-film capacitors^1^,^2^,^3^,^4^,^5^,^6^,^7^ and transistors^8^,^9^,^10^,^11^,^12^,^13^,^14^,^15^ for memory applications. In 2008, Asadi *et al.* used phase-separated blends of P(VDF-TrFE) and semiconducting polymers to realise a bistable resistive memory diode^16^,^17^,^18^. In this diode, the semiconducting polymer forms a resistive pathway from one electrode to the other that can be modulated via the ferroelectric polarisation of P(VDF-TrFE). This diode combines the appealing features of P(VDF-TrFE) with those of a memory diode (i.e., rectification and non-destructive resistive readout), thereby enabling simple yet high-density crossbar array integration^16^,^17^,^18^,^19^,^20^. Recently, a 32 × 32 crossbar array has been fabricated using these bi-stable diodes^21^. In this 1-kb array, each crossing of electrode lines defines a memory cell that can store a single bit of information.

The inherent problem with low-cost processing is the resulting relatively low areal density. This drawback can be partially mitigated by exploiting the fact that ferroelectric materials generally offer the capability of partial switching, enabling the storage of multiple bits per memory element^22^,^23^,^24^. However, multilevel data storage generally requires either complex addressing methods, in the form of voltage or current modulation, or additional complex processing steps^13^,^22^,^23^,^24^. This study presents a novel multi-bit scheme that allows for non-destructive readout and simple pulsed writing; this is made possible via a thorough characterisation of the dynamic response. The switching time depends exponentially on the applied electric field, and the coercive fields are found to depend on the polarising time. We demonstrate that the ferroelectric response is primarily controlled by a statistical distribution of the local electric field rather than by a temporal law of local polarisation switching, i.e., the electric-field dependence can be explained in terms of the inhomogeneous field mechanism (IFM) model that has been established for a wide range of disordered ferroelectrics^24^,^25^,^26^. These insights introduce the possibility of multilevel polarisation (MLP) states, which are demonstrated in the diodes investigated in this study. Four stable polarisation states can be programmed and read out repeatedly in a single memory diode. The resulting 2-bit data storage per memory element doubles the storage density of the organic ferroelectric resistive memory diode without increasing its technological complexity, thus reducing the cost per bit.

## Results & Discussion

**Figure 1:**
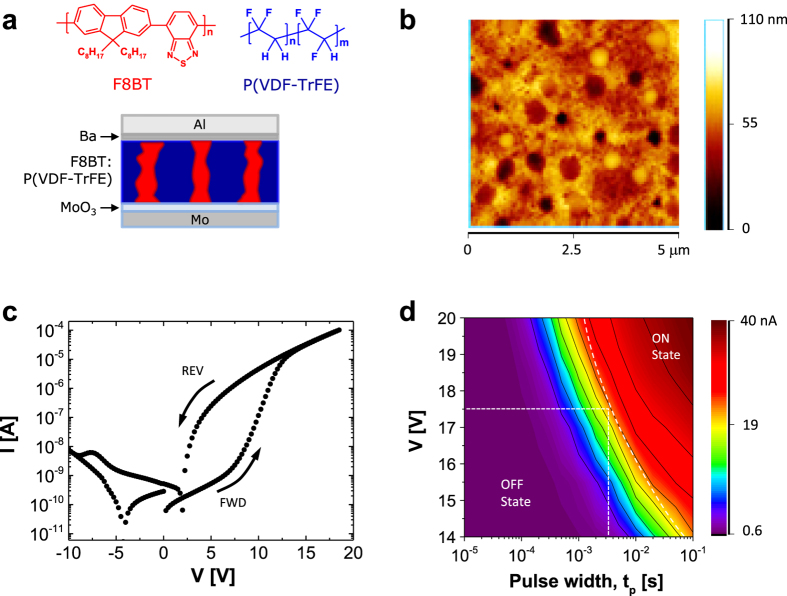
(**a**) Chemical structures of F8BT (red) and P(VDF-TrFE) (blue) and a schematic illustration of a polymer ferroelectric resistive memory cell; (**b**) AFM topological image of a P(VDF-TrFE):F8BT (9:1 (w/w)) blended film on an MoO_3_/Mo electrode, and (**c**) static I-V characteristics and (**d**) dynamic characteristics of the ferroelectric memory diodes. The colour scale indicates the device ON current measured at 5 V after switching pulses of different pulse widths (i.e., times) and pulse heights (i.e., voltages).

Figure 1a schematically illustrates the structure of our organic ferroelectric resistive memory device and the materials used. Poly(9,9-di-n-octylfluorene-alt-benzothiadiazole), F8BT, is an ambipolar semiconductor whose charge transport has been well characterised in diodes and transistors^27^,^28^,^29^. P(VDF-TrFE) is a wide band-gap insulator, and its ferroelectric properties have been well described^1^,^2^,^3^,^4^,^5^,^6^,^30^,^31^,^32^,^33^. A 1:9 (w/w) polymer blend solution of F8BT and P(VDF-TrFE) was spin coated onto glass substrates with patterned Mo/MoO_3_ electrodes; these substrates were subsequently annealed at 135 °C for 1 hour to enhance the crystallinity of the ferroelectric phase. For more processing details, see the Experimental section and previously published works^16^,^17^,^18^,^19^,^20^. Figure 1b presents an AFM topography image of a 200-nm-thick continuous film. Circular domains of F8BT with diameters of 200–600 nm are surrounded by a P(VDF-TrFE) matrix. Similar phase-separated morphologies have been reported by several groups^20^,^34^,^35^,^36^. In these studies, a wide range of different polymers and small-molecule semiconductors have been used, illustrating that this type of lateral phase-separated morphology, which is key to device operation, is not specific to any one type of semiconductor^7^,^36^. When such a thin film is sandwiched between two metal electrodes, the transport of charge carriers is possible via the semiconducting phase. The work function(s) of the metal contact(s) is (are) purposely chosen to yield substantial injection barrier(s) into the semiconductor. F8BT forms a (hole) injection barrier of ~0.3 eV with Mo/MoO_3_ if the work function for MoO_3_ is taken to be ~5.6 eV and the HOMO level of F8BT is taken to be 5.9 eV^26^,^30^,^31^. The work function of the other Ba/Al electrode (2.8 eV) matches the LUMO energy of F8BT (3.3 eV); an ohmic contact for electron injection is thus expected^21^. When the ferroelectric material is polarised in the correct direction, the polarisation field of the ferroelectric material lowers the injection barrier at the semiconductor-metal interface, and a higher device current is observed^7^,^34^; however, when the ferroelectric material is poled in the other direction, the current is low. A bi-stable resistive switch is thus obtained. A representative quasi-static current versus voltage hysteretic sweep of the ferroelectric memory diodes is presented in Fig. 1c. In the forward voltage scan, the electron (i.e., minority) current is low despite the ohmic contact, whereas the hole (i.e., majority) current is initially limited by the Schottky barrier between MoO_3_ and F8BT, and thus, a relatively low current of 0.52 nA at 5 V is measured. When the applied voltage exceeds the coercive electric field of the ferroelectric P(VDF-TrFE) layer, the injection barrier of MoO_3_/Mo is lowered, and the hole current is significantly increased; the current then reads 0.24 *μ*A at 5 V. Thus, the current ratio for the two polarisation states is ~450, which is sufficiently large for crossbar integration.

To enable controlled partial switching, additional knowledge of the switching kinetics is required. To accomplish this goal, pulsed switching studies were performed (Fig. 1d), during which the duration and magnitude of the programming pulse were varied. Before each programming pulse, the memory was reset to the OFF state by applying a negative pulse of −20 V for 50 ms. Figure 1d presents the results obtained when switching the memory from the OFF to the ON state. The memory remained in the OFF state when short pulses of insufficient amplitude were applied. For large pulses of 20 V, switching to the ON state occurred within 0.2 ms, whereas for lower voltages (i.e., ~14 V), the switching time was near 10 ms. Such a strong electric-field dependence has been previously reported for P(VDF-TrFE) capacitors^3^,^4^,^5^.

**Figure 2:**
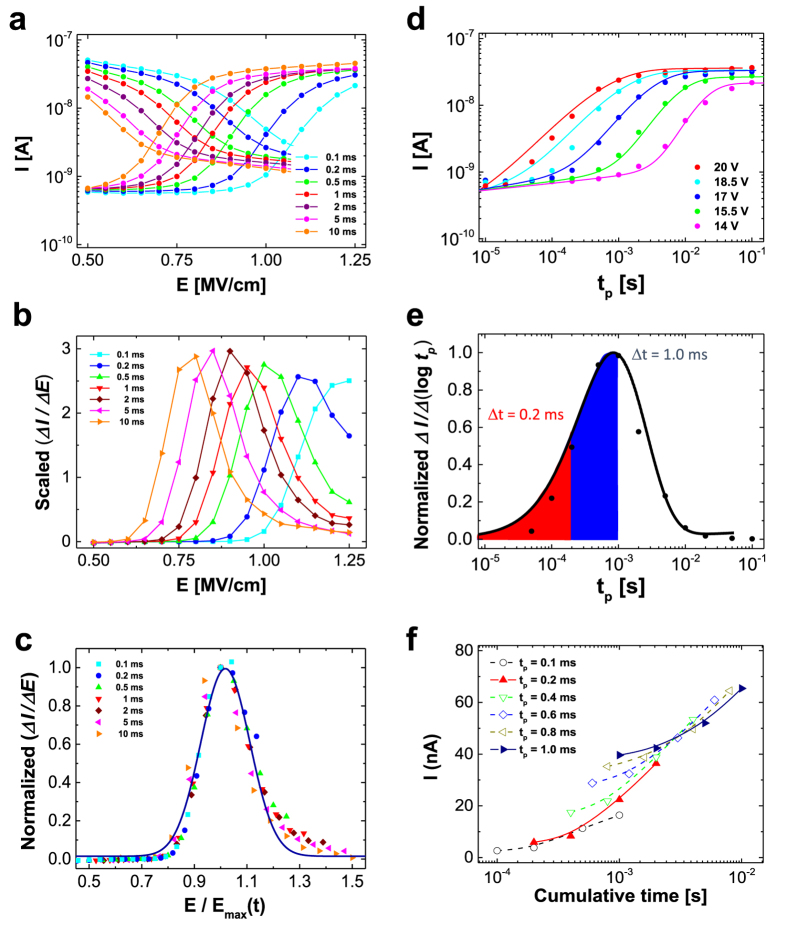
(**a**) Measured currents of the ferroelectric memory diode at 5 V after switching from OFF to ON (i.e., curves starting low) or ON to OFF (i.e., curves starting high) vs. the applied electric field at various pulse widths. (**b**) Scaled derivatives versus the applied field at various pulse widths. (**c**) Normalised curves of (**b**) on an appropriately scaled electric-field axis at the maximum position, *E*_*max*_ (*t*). (**d**) Currents through the organic ferroelectric memory diode after switching from OFF to ON vs. the programming pulse time under various applied fields. The scattered points were measured at 5 V after programming, and the solid lines represent fits to the IFM model. (**e**) Normalised logarithmic derivative curves versus pulse widths at a fixed programming voltage of 18.5 V (*E*_*c*_ = 0.93 MV/cm), where the solid line represents the fitted master curve. (**f**) Device currents attributable to the cumulative effect of successive programming pulses (OFF to ON) at a constant electric field value (*E*_*c*_ = 0.93 MV/cm).

Figure 2a presents the measured currents as functions of the applied field for both the ON-to-OFF transition and the OFF-to-ON transition. The ON-to-OFF transient arises at slightly lower electric fields (i.e., is ‘faster’) than the OFF-to-ON switching transient. We note that symmetric behaviour is not expected a priori, as this would imply a nearly linear relationship between the actual device current and the remanent polarisation. Figure 2b displays the derivative of the memory current vs. the electric field (i.e., ΔI/ΔE) for the OFF-to-ON transition as a function of the applied field for various pulse durations. If the switching time is defined as the time at which ΔI/ΔE peaks, then the switching times can be fit using the following empirical formula^37^:


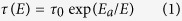


where τ_*0*_ is the characteristic time parameter and *E*_*a*_ is the activation field. The obtained fit values were τ_0_ = ~6.7 ns and *E*_*a*_ = 11.37 (±0.32) MV/cm (please refer to Figure S3 in the Supporting Information for more information). The extracted value of *E*_*a*_ of 11.37 (±0.32) MV/cm is comparable to the value of ~10 MV/cm that has been previously measured in P(VDF-TrFE) capacitors^24^, indicating that the field dependence in both types of memory devices is similar. The longer switching times measured for our devices are related to the exponential pre-factor. The τ_*0*_ value of ~6.7 ns in the diodes is somewhat larger than the previously reported value of ~1 ns in the capacitor structure^26^. Typically, the τ_*0*_ parameter is associated with the accumulation of screening charges at the interfaces between non-ferroelectric and ferroelectric layers. Hence, a larger τ_*0*_ value suggests that charge carriers flow via the non-ferroelectric regions of the film (e.g., the amorphous phase of the grain boundaries in the P(VDF-TrFE) matrix or the polymer semiconductor pillars interfacing with the P(VDF-TrFE) matrix) and form an interfacial charge layer at the phase boundaries^26^.

Strikingly, all ΔI/ΔE curves display a similar shape, with a characteristic width and height. This observation can be explained by the IFM model^24^,^25^,^26^. In this model, a ferroelectric material is considered to consist of randomly distributed regions with independent polarisation kinetics governed by a switching-time-dependent local field. In other words, the time-dependent local field depends on the domains that have and have not switched before. Therefore, if the kinetic process is deterministic, the various domains will always switch in the same order, and their transients can be expected to exhibit a universality that can be expressed as a master curve. Indeed, the properties of the derivatives presented in Fig. 2b allow us to extract such a master curve through the Gaussian fitting of the normalised data points, as shown in Fig. 2c. Details regarding the extraction of the IFM master curve are provided in the Supporting Information.

Until recently, the applications of this new type of device have been restricted to only the two saturated values. In principle, it is possible to obtain an intermediate current between the saturated values by adjusting the ratio of upward to downward polarisation. However, the achievement of an intermediate state with the desired polarisation value is challenging because of the stochastic and complex nature of ferroelectric polarisation switching and possible depolarisation effects. Our devices, however, can be well described in the framework of the IFM model, and hence, we attempted to store multiple bits in a single memory cell by polarising only certain ferroelectric regions instead of all of them^13^,^22^,^24^. Figure 2d presents the transient current curves as a function of the switching pulse time, where the scattered points represent measured data and the lines represent fits to the IFM model. The shape of the fitted transient curve vs. the logarithm of the switching pulse time is similar to that of the transient current curves vs. the electric field, as observed in Fig. 2a. This observation suggests that it may be informative to differentiate the fitted transient curves with respect to the logarithm of the pulse time; the resulting Gaussian distribution is presented in Fig. 2e. The kinetics of the independent local polarisation in P(VDF-TrFE) ferroelectrics follow a stretched exponential dependence on the switching pulse time^24^,^25^. Therefore, conceptually, partial polarisation in a ferroelectric matrix can be modulated by adjusting both the switching time and the electric field. Figure 2f illustrates the feasibility of pulse-modulated multilevel data storage in organic ferroelectric memory, where intermediate current states can be reliably programmed. The curves highlight that adjustments to the switching time and electric field are, to a large degree, interchangeable, as the same state can be achieved in multiple ways.

For the actual demonstration of 2-bit data storage in an organic ferroelectric diode cell, we selected two intermediate states with programming times of 0.2 ms and 1 ms in addition to the complete OFF and complete ON states, each of which had a programming time of 50 ms (see Figure S4 in the Supporting Information). The measured currents at 5 V after programming pulses of different durations and equal electric field strength took on discrete values for corresponding pulse durations. Furthermore, the readout current after a number of successive programming pulses was less than that after a single pulse of the corresponding cumulative duration because the memory current is exponentially related to the pulse duration.

**Figure 3:**
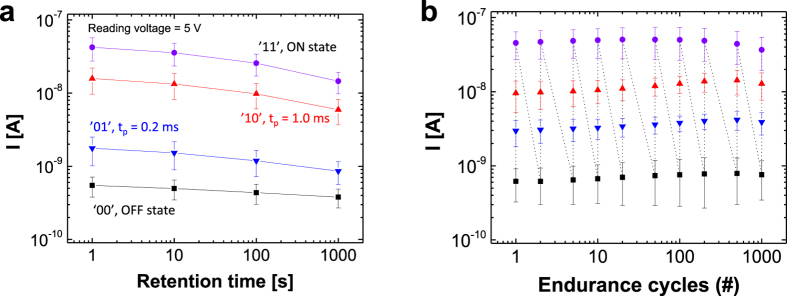
(**a**) Retention time of programmed multilevel data and (**b**) multiple write/erase endurance cycles of multilevel data stored in the organic ferroelectric resistive memory diode.

The memory reliability of pulse-modulated multilevel data storage in the organic ferroelectric memory switch was examined in terms of both time-dependent data retention and endurance over multiple instances of data programming/erasing. Data retention was evaluated by independently measuring four data storage levels controlled by different pulse-switching times at a fixed programming voltage of 18.5 V (E_c_ = 0.93 MV/cm). Figure 3a reveals reasonably reliable data retention of all four states for up to approximately 10^3^ s. It should be noted that the charge retention of this multilevel ferroelectric memory device is relatively short, which may be attributable to the partial depolarisation of ferroelectric components interfacing with semiconducting polymer pillars; this behaviour is significantly different from that of capacitance-type organic ferroelectric memory, and thus, further improvements should be sought to increase the retention time, including the optimisation of semiconducting components and/or a more careful selection of electrodes. However, it is notable that the retention of the intermediate states is comparable to or even better than that of the normal operation mode (cf. the upper ‘ON’ curve). Next, for measurements of endurance under multiple data-writing cycles, we consecutively erased/wrote each of the four levels of data storage (i.e., ‘00,’ ‘01’, ‘10’, and ‘11’ states) and read the current after n iterations of this programmed endurance cycle (n = 1, 2, 5, 10, … 500, 1000). Very consistent and reliable rewritability of all four levels was observed for over 1000 cycles, as shown in Fig. 3b. The results indicate that the degree of ferroelectric polymer domain switching was precisely controlled and remained unchanged when a proper combination of pulse duration and voltage was repeatedly applied.

**Figure 4:**
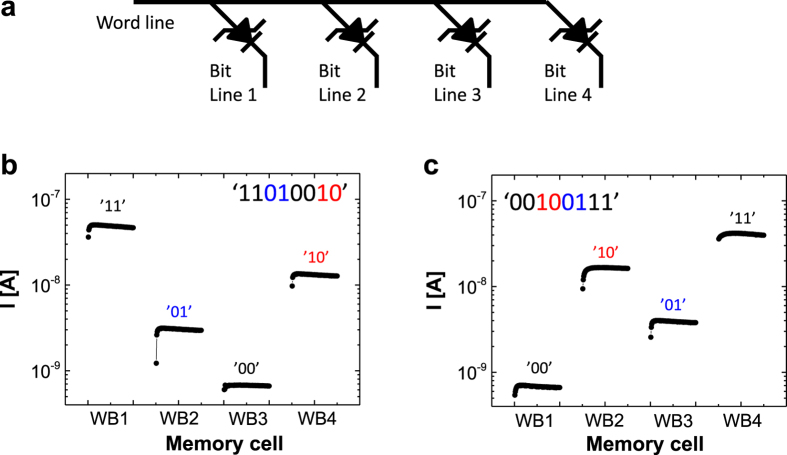
(**a**) Schematic representation of the 1 × 4 organic ferroelectric crossbar memory diode array, and the readout currents for 8-bit logic data stored in the 1 × 4 memory array: (**b**) ‘11010010’ and (c) ‘00100111’.

Finally, to demonstrate the feasibility of doubling the information density of a real memory device using pulse-modulated MLP states, we tested a simple 1 × 4 organic ferroelectric crossbar memory diode array, wherein the word line (WL) was common and the bit line (BL) was separated (Fig. 4a). To write an example state of ‘11010010’ into the 1 × 4 crossbar memory array, different programming pulse widths of 50 ms, 0.2 ms, 0 ms, and 1 ms at a fixed programming voltage of 18.5 V, corresponding to ‘11’, ‘01’, ‘00’, and ‘10’, respectively, were sequentially applied to each bit line. Then, in the same sequence in which the data were written, a fixed current-measuring voltage of 5 V was applied to non-destructively read the stored multilevel data. Figure 4b clearly reveals that discrete and distinct current levels corresponding to the specific pulse widths in each memory cell could be read out. In a similar manner, we wrote and read the 8-bit state ‘00100111’ into the 1 × 4 crossbar memory array, as shown in Fig. 4c. Both examples successfully demonstrated that the memory density of the simple organic ferroelectric crossbar memory diode array could be reliably doubled by the controlled programming pulse width, as intended.

## Conclusion

In summary, we demonstrated the capability of pulse-modulated multilevel data storage in organic ferroelectric resistive memory diodes. The principle can be understood based on an investigation of the dynamic operating principles of the organic memory device. The dynamic switching mechanism of our organic resistive memory can be explained by the IFM model, in which the ferroelectric components are regarded as an assembly of randomly distributed regions with independent polarisation kinetics governed by a time-dependent local field. Therefore, we could reliably partially polarise the ferroelectric material using tuned programming pulses to achieve 2-bit data storage in a single organic ferroelectric diode cell with stable retention and endurance. We believe that these findings lay the foundations for organic ferroelectric memory devices that offer higher storage density without increased technological complexity.

## Methods

For device fabrication, the bottom anode layers were initially formed on glass substrates through the e-beam evaporation of Mo, the subsequent deposition of MoO_3_, and finally, patterning using standard photolithography. Next, a 200-nm-thick continuous film of a 1:9 (w/w) blend of F8BT and P(VDF-TrFE), with 77 mol% VDF, was spun in an initially homogeneous evaporative solution, followed by thermal annealing at 135 °C for 1 hour to facilitate the growth of the ferroelectric ß phase. The decomposition of the mixed polymer gave rise to a dispersed phase consisting of pillar-like semiconductor domains embedded in a ferroelectric matrix. The Ba/Al top cathodes were evaporated through a shadow mask, and finally, the devices were encapsulated with a conformable SiN_x_ barrier layer to avoid charge trapping at the interfaces between the electrodes and the polymer semiconductors, which can affect the multilevel resistive switching phenomenon. A schematic illustration of this organic ferroelectric resistive memory device is presented in Fig. 1a. A commercial AFM system (Veeco MultiMode NS-IIIA) with Al-coated Si tips (Nanosensors, spring constant k = 12.5 N/m) was used to characterise the blend structure at the nanoscale. Electrical measurements were performed at room temperature in ambient conditions using an Agilent 33120A function generator (i.e., the source) in combination with an external voltage amplifier, a Keithley 6485 picoammeter (i.e., the sensor), and two Keithley 2612A source meters controlled by a homebuilt “FerrOrgan” switching box.

## Additional Information

**How to cite this article**: Lee, J. *et al.* Pulse-modulated multilevel data storage in an organic ferroelectric resistive memory diode. *Sci. Rep.* **6**, 24407; doi: 10.1038/srep24407 (2016).

## Supplementary Material

Supplementary Information
