# Supplementary material for: Pulse-modulated multilevel data storage in an organic ferroelectric resistive memory diode
Source: Sci Rep. 2016 Apr 15;6:24407. doi: 10.1038/srep24407 (PMC4832143; doi:10.1038/srep24407)
Supplement: Supplementary Information [file srep24407-s1.pdf]

Supplementary Information

Pulse-modulated multilevel data storage in an organic ferroelectric resistive memory diode

Jiyoul Lee[1,2,*], Albert J.J.M. van Breemen[1], Vsevolod Khikhlovskyi[1, 3], Martijn Kemerink[1,4], Rene A.J. Janssen[3], Gerwin H. Gelinck [1,3,*]

[1]*Holst Centre/TNO, High Tech Campus 31, 5656 AE Eindhoven, The Netherlands*
[2]*Department of Graphic Arts Information Engineering, Pukyong National University, 608-739, Busan, S. Korea*
[3] *Department of Applied Physics, Eindhoven University of Technology, P. O. Box 513, 5600 MB Eindhoven, The Netherlands*
[4]*Department of Physics, Chemistry and Biology (IFM), Linköping University, SE-58183, Sweden*

* jiyoul_lee@pknu.ac.kr or gerwin.gelinck@tno.nl

Polarisation characteristics of P(VDF-TrFE) ferroelectric films

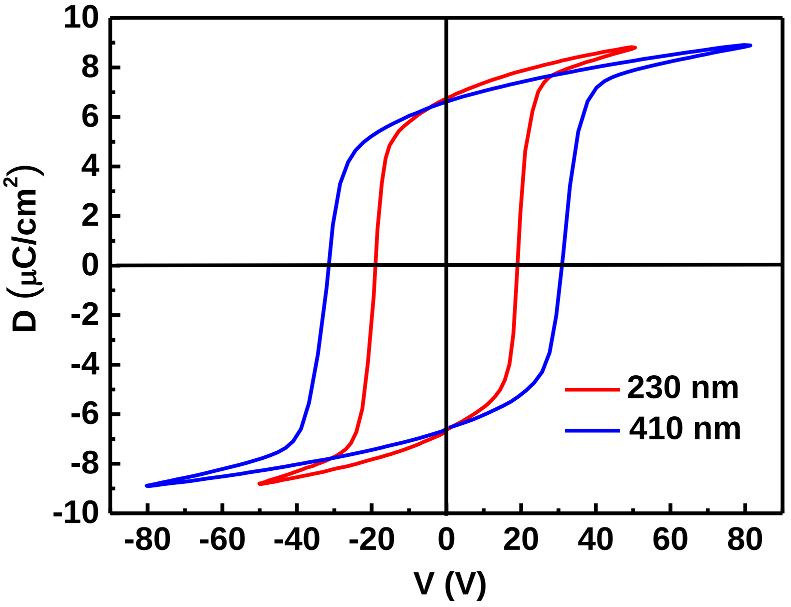

Figure S1. Ferroelectric hysteresis loops of P(VDF-TrFE) films with different layer thickness.

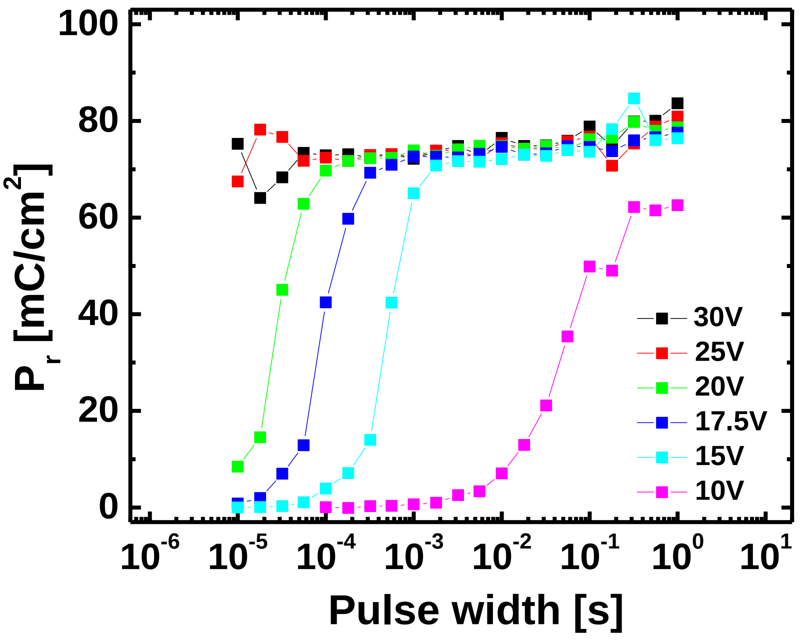

Figure S2. Pulse width dependence of polarisation of a ~200 nm thick P(VDF-TrFE) films at various pulse voltages. The Positive-Up-Negative-Down (PUND) measurement results show the switching characteristics of the P(VDF-TrFE) capacitor device.

Extraction of the master curve using the inhomogeneous field mechanism (IFM) model

According to the IFM model[1~3], intrinsically inhomogeneous ferroelectric materials such as P(VDF-TrFE) are divided into randomly distributed regions with independent polarisation kinetics governed by a time-dependent local electric field. In fact, the IFM model is not strictly applicable to an organic ferroelectric memory diode because of the non-linear dependence between the actual memory current and the net remanent polarisation. However, because the memory currents are still monotonically dependent on the polarisation in the ferroelectric component, with careful scaling of each function, we can nevertheless apply the IFM model. Therefore, the extraction of the IFM master curve for the organic ferroelectric memory diode must begin with the assumption that $\Delta I \propto \rho(\Delta P)$, where $\rho(x)$ is a non-linear function, such as an exponential function.[4] Based on the time-dependent changes in polarisation, the total polarisation of the P(VDF-TrFE) matrix, $\Delta P(E_m, t)$, can be determined as follows:[1~3]

$$\Delta P(E_m,t) = \int_0^\infty g(E_m,\tau)\cdot p(t,\tau)\,d\tau = \int_0^\infty E_m^{-1} f(E/E_m)\cdot p(t,\tau(E))\,dE\ , \qquad \text{(S1)}$$

where E_m is the mean applied electric field, defined as $V(t)/d$, where d is the film thickness; $p(t, \tau(E))$ represents the stretched exponential dependence of the local polarisation, with some characteristic time τ; and τ can be expressed by the well-known empirical function $\tau(E) = \tau_0 \exp\left(E_a / E\right)$, where τ_0 is the characteristic time parameter and E_a is the activation field.[5] The statistical distribution functions of the switching time $g(E_m, \tau)$ and the local field amplitude $f(E/E_m)$ are related by $E_m^{-1}\cdot f(E/E_m) = g(E_m, \tau)\cdot d\tau/dE$.[1, 2]

We proceed by approximating the function p as a Heaviside step function in time, $\Delta P_{max}\cdot\theta[t - \tau(E)]$, or in the field, $\Delta P_{max}\cdot\theta[E - E_{th}(t)]$, where τ is defined by the inflection point of the actual function $p(t, \tau(E))$ on the logarithmic time scale and the threshold field $E_{th}(t)$ results from the solution of $t = \tau(E)$.[1] Then, the polarisation $\Delta P(E_m, t)$ of the P(VDF- TrFE)

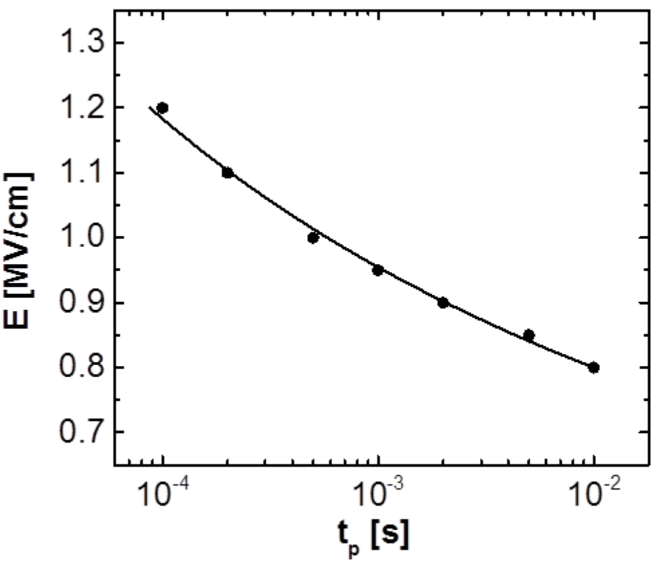

Figure S3. Switching pulse time vs. electric field to determine τ_0 and E_a of the empirical function, as suggested by W. J. Merz [5]. The symbols represent measured data, and the solid line represents the fit to the function.

matrix in equation S1 results from the integration of the distribution function from $E_{th}(t)$ to infinity:

$$\Delta P(E_m, t) \cong \Delta P_{\max} \int_{E_{th}(t)}^{\infty} \frac{dE}{E_m} f\left(\frac{E}{E_m}\right). \qquad \text{(S2)}$$

If we differentiate equation S2 with respect to the logarithm of E_m, the following equation can be obtained:

$$\frac{\partial \Delta P(E_m, t)}{\partial \ln E_m} = \Delta P_{\max} \frac{E_{th}(t)}{E_m} f\left(\frac{E_{th}(t)}{E_m}\right). \qquad \text{(S3)}$$

The right-hand side of this equation is a function of the combined variable $E_m/E_{th}(t)$; thus, the left-hand side must also be a function of such a variable.[1, 2] All logarithmic derivatives exhibit a maximum at some time-dependent position $E_{max}(t)$, as shown in Figure 2b of the main text. Equation S3 implies that the experimental dependencies of the logarithmic derivative on the applied field at different times should exhibit the same shape, reproducible from the same master curve, $\Phi[E_m/E_{max}(t)]$, where $E_{max}(t)$ is the position of the maximum of this derivative at time t (see Figure 2c). Thus, $E_{max}(t) = \gamma E_{th}(t)$ should be satisfied with a constant γ. Then,

equation S3 allows for the explicit determination of the function $f(x)$ from the master curve, $f(x) = 1/x \cdot \Phi[1/\gamma x]$, and the constant γ is also strictly determined by $\gamma = \int_0^\infty \frac{dx}{x^2}\Phi(x)$.[1]

Intermediate states of the memory current

Discrete levels of memory currents modulated by pulsed switching were examined as a function of the programming pulse time; these results are presented in Figure S4. There are many intermediate states available, which may allow for a three- or even four-fold increase in memory density. To ensure the reliability and robustness of multilevel data storage in the cell, however, we selected only two different readout current levels, with a sufficient margin to allow for deviating readout currents, and set each level via a corresponding programming pulse width.

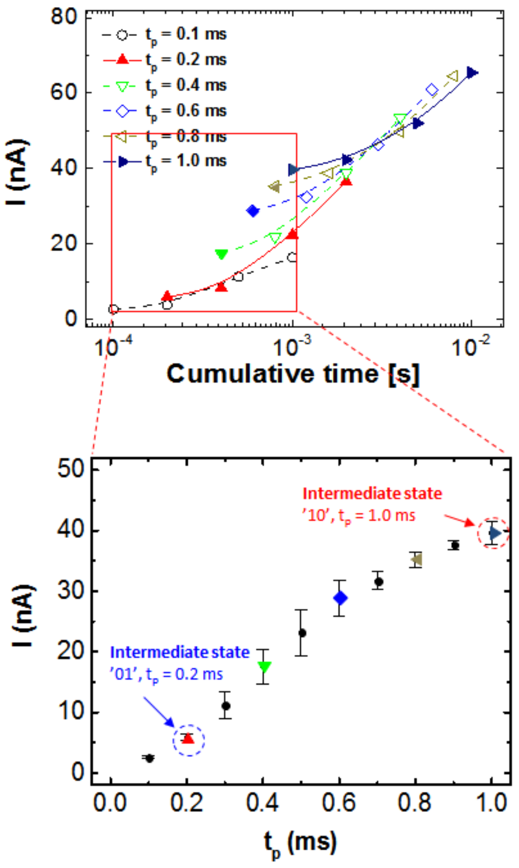

Figure S4. Linearly scaled plot of the readout currents at a reading voltage of 5 V vs. the width of a single programming pulse at a programming voltage of 18.5 V. This plot is a magnified view of the indicated region of Figure 2f.

Device reliability test results

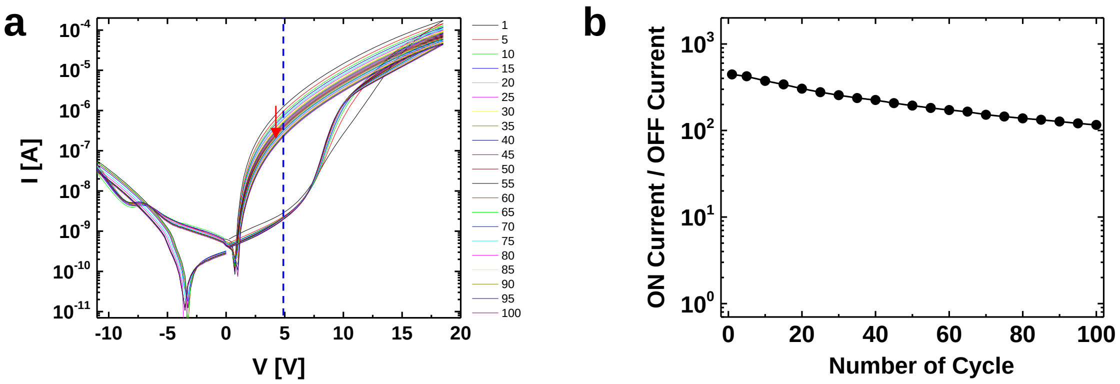

Figure S5. Device reliability test results of the organic memory diodes. (a) Cycling I-V curve and (b) the ON/OFF current ratios at 5V of the organic memory didoes for 100 times. For 100 cycles, the ON-OFF current ratio is sustained to over two orders of magnitude.

References

1. Zhukov, S., Genenko, Y. A., Hirsch, O., Glaum, J., Granzow, T., & von Seggern, H. Dynamics of polarization reversal in virgin and fatigued ferroelectric ceramics by inhomogeneous field mechanism. *Physical Review B*, *82*(1), 014109. (2010).
2. Schütrumpf, J., Zhukov, S., Genenko, Y. A., & Von Seggern, H. Polarization switching dynamics by inhomogeneous field mechanism in ferroelectric polymers. *Journal of Physics D: Applied Physics*, *45*(16), 165301. (2012).
3. Genenko, Y. A., Zhukov, S., Yampolskii, S. V., Schütrumpf, J., Dittmer, R., Jo, W., Kungl, H., Hoffmann, M. J. & von Seggern, H., Universal polarization switching behavior of disordered ferroelectrics. *Advanced Functional Materials*, *22*(10), 2058-2066. (2012).
4. Kemerink, M., Asadi, K., Blom, P. W., & de Leeuw, D. M. The operational mechanism of ferroelectric-driven organic resistive switches. *Organic electronics*, *13*(1), 147-152. (2012).
5. Merz, W. J., Domain Formation and Domain Wall Motions in Ferroelectric BaTiO3 Single Crystals. *Physical Review*, *95*(3), 690. (1954).
